# Supplementary material for: HIVprotI: an integrated web based platform for prediction and design of HIV proteins inhibitors
Source: J Cheminform. 2018 Mar 9;10:12. doi: 10.1186/s13321-018-0266-y (PMC5845081; doi:10.1186/s13321-018-0266-y)
Supplement: Supplementary file 2 — Additional file 2. Source code of HIVProtI web server. [file 13321_2018_266_MOESM2_ESM.zip › HIVprotI_Source-code/hivprotI/weka-3-6-6/doc/weka/gui/JListHelper.html]

JListHelper


---


|  |  |  |
| --- | --- | --- |
| **Overview** \| **Package** \| **Class** \| **Tree** \| **Deprecated** \| **Index** \| **Help** \| **Weka's home** | |  |
| **PREV CLASS**   **NEXT CLASS** | **FRAMES**    **NO FRAMES**     **All Classes** |  |
| SUMMARY: NESTED \| FIELD \| CONSTR \| METHOD | DETAIL: FIELD \| CONSTR \| METHOD |  |


---


## weka.gui Class JListHelper

```
java.lang.Object
  weka.gui.JListHelper
```

---

``` public class JListHelper extends java.lang.Object ```

A helper class for JList GUI elements with DefaultListModel or
derived models.

**Version:**
:   $Revision: 7059 $

**Author:**
:   FracPete (fracpete at waikato dot ac dot nz)

**See Also:**: `JList`, `DefaultListModel`

---

| **Field Summary** | |
| --- | --- |
| `static int` | `MOVE_DOWN`             moves items down |
| `static int` | `MOVE_UP`             moves items up |


**Constructor Summary**

`JListHelper()`


| **Method Summary** | |
| --- | --- |
| `static boolean` | `canMoveDown(javax.swing.JList list)`             checks whether the selected items can be moved down |
| `static boolean` | `canMoveUp(javax.swing.JList list)`             checks whether the selected items can be moved up |
| `static void` | `moveBottom(javax.swing.JList list)`             moves the selected items to the end |
| `static void` | `moveDown(javax.swing.JList list)`             moves the selected item down by 1 |
| `static void` | `moveTop(javax.swing.JList list)`             moves the selected items to the top |
| `static void` | `moveUp(javax.swing.JList list)`             moves the selected items up by 1 |

**Methods inherited from class java.lang.Object**

`equals, getClass, hashCode, notify, notifyAll, toString, wait, wait, wait`

**Field Detail**

### MOVE\_UP

```
public static final int MOVE_UP
```

:   moves items up

    **See Also:**: Constant Field Values

---


### MOVE\_DOWN

```
public static final int MOVE_DOWN
```

:   moves items down

    **See Also:**: Constant Field Values


**Constructor Detail**

### JListHelper

```
public JListHelper()
```


**Method Detail**

### moveUp

```
public static void moveUp(javax.swing.JList list)
```

:   moves the selected items up by 1

    :   **Parameters:**: `list` - the JList to work on

---


### moveDown

```
public static void moveDown(javax.swing.JList list)
```

:   moves the selected item down by 1

    :   **Parameters:**: `list` - the JList to work on

---


### moveTop

```
public static void moveTop(javax.swing.JList list)
```

:   moves the selected items to the top

    :   **Parameters:**: `list` - the JList to work on

---


### moveBottom

```
public static void moveBottom(javax.swing.JList list)
```

:   moves the selected items to the end

    :   **Parameters:**: `list` - the JList to work on

---


### canMoveUp

```
public static boolean canMoveUp(javax.swing.JList list)
```

:   checks whether the selected items can be moved up

    :   **Parameters:**: `list` - the JList to work on

---


### canMoveDown

```
public static boolean canMoveDown(javax.swing.JList list)
```

:   checks whether the selected items can be moved down

    :   **Parameters:**: `list` - the JList to work on


---


|  |  |  |
| --- | --- | --- |
| **Overview** \| **Package** \| **Class** \| **Tree** \| **Deprecated** \| **Index** \| **Help** \| **Weka's home** | |  |
| **PREV CLASS**   **NEXT CLASS** | **FRAMES**    **NO FRAMES**     **All Classes** |  |
| SUMMARY: NESTED \| FIELD \| CONSTR \| METHOD | DETAIL: FIELD \| CONSTR \| METHOD |  |


---
